# Enhancement of select cognitive domains with rosiglitazone implicates dorsal hippocampus circuitry sensitive to PPARγ agonism in an Alzheimer's mouse model

**DOI:** 10.1002/brb3.1973

**Published:** 2020-12-31

**Authors:** IbDanelo Cortez, Caterina M. Hernandez, Kelly T. Dineley

**Affiliations:** ^1^ Department of Psychology University of Houston Houston TX USA; ^2^ Department of Pharmaceutical Sciences Appalachian College of Pharmacy Oakwood VA USA; ^3^ Department of Neurology the University of Texas Medical Branch at Galveston Galveston TX USA

**Keywords:** context discrimination, fear conditioning, hippocampus, learning and memory, Morris water maze, PPARγ

## Abstract

**Introduction:**

Several clinical studies have tested the efficacy of insulin‐sensitizing drugs for cognitive enhancement in Alzheimer's disease (AD) patients, as type 2 diabetes (T2D) is a well‐recognized risk factor for AD. Pilot studies assessing FDA‐approved diabetes drugs in subjects with early‐stage disease have found cognitive benefit in subjects comorbid for insulin resistance. In AD mouse models with concomitant insulin resistance, we have shown that 4 weeks of RSG can reverse peripheral and central insulin resistance concomitant with rescue of hippocampus‐dependent fear learning and memory and hippocampal circuitry deficits in 9‐month‐old (9MO) Tg2576 mice with no effect in wild‐type (WT) mice. Bioinformatics analysis of genomic and proteomic data reveals an intimate link between PPARγ and MAPK/ERK signaling in the hippocampus. We then demonstrated a direct interaction between PPARγ and phospho‐ERK in vitro and in vivo during memory consolidation. The translational value of this discovery is evidenced by the positive correlational relationship between human AD postmortem brain levels of pERK‐PPARγ nuclear complexes with cognitive reserve.

**Methods:**

We tested whether insulin sensitizer therapy could rescue spatial navigation, context discrimination, and object recognition learning and memory in aged wild‐type and Tg2576 mice in addition to hippocampus‐dependent contextual fear learning and memory, as we have previously reported.

**Results:**

We found that rosiglitazone treatment improved cognitive domains that predominantly rely upon the dorsal hippocampus rather than those that additionally engage the ventral hippocampus.

**Conclusion:**

These results suggest that insulin sensitizer therapy with rosiglitazone improved age‐ and AD‐related learning and memory deficits in circuit selective ways.

## Graphical abstract

The dHIP (dorsal hippocampus) and vHIP (ventral hippocampus) are distinct functional structures for learning and memory. The dorsal hippocampus is crucial for spatial navigation within a specific context whereas the ventral hippocampus processes contextual information related to stress, emotion and affect. These functions are supported by circuit connectivity in that the dorsal hippocampus communicates with cortical regions involved in decision‐making and information processing, while the ventral hippocampus communicates with regions involved in emotion and stress (e.g., amygdala and hypothalamus). The learning and memory tasks probed in this study indicate that the Tg2576 Alzheimer’s disease mouse model exhibits both dorsal and ventral hippocampal circuit deficits. PPARγ agonism with RSG (rosiglita zone) enhanced cognitive performance in Tg2576, but treatment effects were restricted to cognitive domains that rely more heavily on dorsal hippocampus.

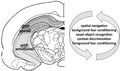

## 1 INTRODUCTION

Alzheimer's disease (AD), the most common form of dementia, is a progressive neurodegenerative disease that is characterized by progressive memory decline, brain atrophy, and the presence of misfolded proteins: amyloid beta plaques and tau fibrillary tangles in the brain (Serrano‐Pozo et al., 2011). Millions of new patients around the world are diagnosed with AD every year, and with no cure or disease‐modifying treatment available, these numbers are expected to climb with the potential to devastate elder populations, particularly in developed countries (The Alzheimer's Association, 2019). As such, much attention has pivoted toward identifying lifestyle risk factors and comorbidities for AD. Type 2 diabetes (T2D) is one such comorbid risk factor that also is on the rise (Jayaraman & Pike, 2014). In fact, the risk of developing AD is 1.5‐ to 2‐fold higher in patients with T2D (Cheng et al., 2012; Janson et al., 2004; Luchsinger et al., 2004; Willette et al., 2015). Furthermore, impaired central insulin signaling manifests similar pathology to that of early AD including neuroinflammation accompanied by deficits in synaptic plasticity and episodic memory (Blazquez et al., 2014; Srodulski et al., 2014), suggesting overlapping mechanisms of central insulin resistance and early AD‐like cognitive impairment (Bedse et al., 2015; Watson & Craft, 2003). In fact, the insulin receptor and its activity are significantly compromised in postmortem hippocampus and cortex of subjects with mild cognitive impairment or early AD (Craft & Watson, 2004; Talbot et al., 2012; Watson et al., 2005).

The T2D drugs, rosiglitazone (RSG, trade name Avandia®) and pioglitazone (trade name Actos™), are members of the thiazolidinedione (TZD) drug class that improve insulin sensitivity in T2D patients (PubChem, 2005). TZDs target the nuclear receptor and transcription factor, peroxisome proliferator‐activated receptor gamma (PPARγ), to induce a gene expression profile that promotes improved glucose metabolism and insulin sensitivity.

Similar to humans with T2D, several AD mouse models also exhibit glucose dysregulation, hyperinsulinemia, and insulin resistance; we have demonstrated this precisely in the Tg2576 mouse model, and the ability of TZDs to ameliorate insulin resistance and associated abnormalities and enhance cognition in this model (Dineley et al., 2014; Lee et al., 2018; Rodriguez‐Rivera et al., 2011; Velazquez et al., 2017). In our hands, RSG improves hippocampus‐dependent memory concomitant with normalized hippocampal circuit function in aged mice and the Tg2576 AD mouse (Cortez et al., 2019; Denner et al., 2012; Jahrling et al., 2014; Nenov et al., 2014, 2015; Rodriguez‐Rivera et al., 2011). Bioinformatics analysis of genomic and proteomic data reveals an intimate link between PPARγ and MAPK/ERK signaling in the hippocampus (Denner et al., 2012). More recently, we have demonstrated a direct interaction between PPARγ and phospho‐ERK in vitro and in vivo during memory consolidation (Jahrling et al., 2014). Thus, our laboratory discovered a dynamic PPARγ and phospho‐ERK network important for rescuing hippocampus‐dependent learning and memory in preclinical models. The translational value of this discovery is evidenced by the positive correlational relationship between postmortem brain levels of pERK‐PPARγ nuclear complexes with AD cognitive reserve (Jahrling et al., 2014).

In late‐onset AD (LOAD), genetic and environmental risk factors are thought to contribute to synaptic loss, and initiation of progressive memory deficits triggered by aberrant amyloid beta (Aβ) accumulation (Golde et al., 2011; Jack et al., 2011; Sperling et al., 2011). The Tg2576 model overproduces Aβ (APP_695_K_670_L/M_671_N) and recapitulates hippocampus‐dependent memory impairment that is common to human LOAD (Alvarez et al., 2008; Dineley et al., 2007; Hoefer et al., 2008; Hort et al., 2007; Kawarabayashi et al., 2001; Kobayashi & Chen, 2005; Sabbagh et al., 2013; Snellman et al., 2013; Westerman et al., 2002). Further, there is extensive literature showing that the mouse phenotype overlaps with currently accepted biomarkers for early AD etiology in humans (Kawarabayashi et al., 2001; Sabbagh et al., 2013; Snellman et al., 2013; Westerman et al., 2002). Albeit Tg2576 is limited as a model for the full spectrum of AD etiology (Ashe & Zahs, 2010), it has utility for modeling the earliest stages of AD due to aberrant Aβ accumulation, the biomarker that distinguishes AD from other dementias. In fact, at 5 months old (MO) Tg2576 exhibit synaptic loss, deficits in contextual fear conditioning, object recognition, and spatial navigation, while Aβ plaques are not observable until 11MO (Dineley et al., 2007; Jacobsen et al., 2006; Jahrling et al., 2014; Rodriguez‐Rivera et al., 2011; Taglialatela et al., 2009). We have shown that 4 weeks of RSG can reverse peripheral and central insulin resistance concomitant with rescue of associative fear memory and hippocampal circuitry deficits in 9‐month‐old (9MO) Tg2576 mice (Jahrling et al., 2014; Rodriguez‐Rivera et al., 2011).

Here, we asked whether RSG could rescue spatial navigation, context discrimination, and object recognition learning and memory deficits when mice are treated from 8 to 9MO. These tasks are hippocampus‐dependent, either exclusively or in part, we do not know the extent to which RSG can improve these cognitive modalities. After 30 days of RSG treatment, we administered novel object recognition (NOR) and Morris water maze (MWM) assays (Hsiao et al., 1996; Taglialatela et al., 2009; Westerman et al., 2002). A separate cohort of 9MO mice were subjected to foreground fear conditioning paradigm, to test the hypothesis that 9MO Tg2576 mice are able to recall contextual conditioning when a discrete auditory stimulus is not paired with the footshock. Lastly, in a third cohort, 9MO WT and Tg2576 mice underwent context discrimination training and testing, as previously described (Cortez et al., 2017), to determine whether a more difficult contextual learning paradigm reveals deficits that may or not be alleviated with RSG PPARγ agonism. We found that RSG treatment improved cognitive domains that predominantly rely upon the dorsal hippocampus rather than encompassing ventral hippocampus. These results suggested that PPARγ agonism affected age‐ and AD‐related learning and memory deficits in circuit selective ways.

## 2 METHODS

### 2.1 Animals

Animals were bred by mating heterozygous Tg2576 males with C57BL6/SJL (F1) females (Jackson Laboratory). The University of Texas Medical Branch operates in compliance with the United States Department of Agriculture Animal Welfare Act, the Guide for the Care and Use of Laboratory Animals, and IACUC‐approved protocols. Animals were pseudorandomly assigned to experimental groups such that each group contained roughly equivalent numbers of males and females, WT and transgenic. Pilot studies determined that male and female Tg2576 mice perform similarly (however see Schmid et al., 2019). Some animals expired during the study unrelated to treatment that affected final animal numbers in some cohorts. Animal numbers are detailed below. Experimenters were blinded to treatment and genotype during key data acquisition and analysis steps.

### 2.2 Insulin sensitizer treatment

Cohorts containing male and female 8MO Tg2576 and WT littermates were randomly assigned to groups fed control (CTRL) or 30mg RSG per kg standard rodent chow (Bio‐Serve) for 30 days, as previously described (Denner et al., 2012; Jahrling et al., 2014; Rodriguez‐Rivera et al., 2011).

### 2.3 Morris water maze

Two cohorts of mice were tested for spatial navigation learning and memory using methods based on Westerman et al. (2002) by an experimenter blinded to genotype and treatment. Four groups (up to 28 mice/group) consisting of Tg2576 and WT littermates either untreated or treated with RSG for 1 month. Water maze testing was conducted in a circular pool (dimensions: 1 m diameter, 75 cm height; water height = 36 cm) made of white, opaque plastic (Coulbourn Instruments). Water was made opaque by the addition of nontoxic white tempera paint (16 oz per fill) and maintained at 25°C ± 2.0°C. Extra‐maze cues (high‐contrast geometric images) were adhered to black nonreflective curtains to hide the experimenter. The pool was divided into 4 quadrants, designated NE, NW, SE, and SW, and 4 release points were centered between quadrants (N, S, E, and W). The pool was sanitized and water refreshed daily prior to each testing session.

#### 2.3.1 Visible platform task

The pool was filled to the surface of a 35‐cm high visible platform (square, 14 cm^2^). The platform was flagged with a dark plastic block (3 × 3 × 9‐cm) to ensure visibility. Daily visible platform training blocks (4 trials/day) were conducted for three consecutive days for cohort 1 and 1 day for cohort 2 since 1 day of visible platform training proved to be sufficient. For each trial, the platform locations (NE, NW, SE, and SW) and release points (centered between quadrants, N, S, E, and W) were pseudorandomized for each trial. The mouse was allowed 60 s to locate the platform. If unsuccessful within the allotted time period, the mouse was directed toward the platform using a dark narrow rod and allowed a 30‐s rest period before the next trial. Following the 4th trial, the mouse was retrieved from the platform and placed on a warming blanket until dry and then replaced into its home cage.

#### 2.3.2 Hidden platform task

On the day following visible platform, daily hidden platform training blocks (4 trials/day) were conducted for nine consecutive days. In contrast to visible platform tests, the platform location was stationary and centered within the SE quadrant and hidden from visibility by submersion ~1 cm below the water line. Mice were released from points centered at quadrant walls, excluding the SE quadrant with the hidden platform. Each mouse had a single training block per day. A training block was defined as four consecutive trials, and the intertrial interval was 30 s. For each trial, the mouse was allowed to swim a maximum of 60 s to locate the platform. When successful, the mouse was allowed a 30‐s rest period on the platform. If unsuccessful, the mouse was directed toward the platform using a dark narrow rod, allowed a 30‐s rest period.

#### 2.3.3 Probe trials

At the beginning of the 4th and 7th day of hidden platform training and on day 10, a probe trial was conducted to measure spatial memory for the platform location in the target quadrant (SE). The platform was removed, and mice were allowed to search for the platform for 60 s.

#### 2.3.4 Water maze exclusion criteria

An animal was removed from the study if it did not follow the rod to the platform during training. An animal was also removed if it did not exhibit appropriate search behavior (e.g., no swimming activity).

#### 2.3.5 Water maze data analysis

Swimming activity of each mouse was monitored via a Panasonic BP344 camera mounted directly above the pool. Watermaze 3 video‐tracking software (Coulbourn Instruments) was utilized to record, analyze, and export platform search parameters: latency (s), distance traveled (cm), and swim speed (cm/s), as well as the percentage of total time spent in the target quadrant for a probe trial. An assessment of spatial bias was accomplished by measuring the percentage of time spent in the target quadrant and performing one‐sided Student's *t* test against a theoretical mean of 25%. A one‐ or two‐way analysis of variance (ANOVA), with repeated measures when indicated, was used to compare water maze performance (latency, distance, swim speed, and percent time in target quadrant) between genotypes and treatments. The Tukey post hoc analysis was used to perform pairwise analyses; *p* ≤ .05 was considered significant.

### 2.4 Novel object recognition

Each group consisted of 15–25 mice/group to assess object recognition by an experimenter blinded to genotype and treatment. Four groups consisting of Tg2576 and wild‐type littermates either untreated or treated with RSG for 1 month. Based on our previously published protocol (Taglialatela et al., 2009), we tested whether mice were able to discriminate between a familiar and novel object using a 4‐hr intertrial interval.

Object discrimination data analysis: To assess object recognition memory, a discrimination ratio (novel object interaction time/total interaction time) for each mouse was calculated and analyzed. A one‐sided Student's *t* test against a theoretical mean of .5 was used to test for novel object preference; *p* ≤ .05 was considered significant.

### 2.5 Foreground fear conditioning

In a separate cohort containing 15–16 mice per group, 9MO WT and Tg2576 subjects were trained in foreground fear conditioning using a similar 7‐min training paradigm. Each mouse was placed in a fear conditioning chamber (Lafayette Instruments) and allowed to explore for the first 4 min of training. At minutes 4 and 5, a 2‐s 0.5‐mA footshock was delivered from the metal floor grid. Mice were allowed to explore the training context for an additional 2 min and then placed back in their home cages. Contextual fear conditioning testing 24 hr later occurred in the aversive training environment, but no footshock was administered during the 5 min of testing. Total freezing was measured using recordings and FreezeFrame software (Actimetrics). Motion indices were set according to individual freezing observed by experimenter blinded to genotype and treatment.

### 2.6 Fear conditioning context discrimination

A separate cohort that included 15–20 mice/group of 9MO Tg2576 and age‐matched WT littermates were trained and tested in context fear discrimination adapted from Frankland (1998) by an experimenter blinded to genotype and treatment; please refer to Cortez et al. (2017) for material and method details. Briefly, Tg2576 mice were placed into Context A on training day 0 and allowed to freely explore for 298sec at which point a 2‐s 0.75‐mA footshock was delivered; training ended 15 s later. For the next 6 days, subjects were randomly presented with Context A and the highly similar context B where cardboard inserts were added and tactile, olfactory, auditory, and lighting cues were altered. Motion indices were set according to individual freezing observed by experimenter blinded to genotype and treatment.

## 3 RESULTS

### 3.1 Morris water maze

**FIGURE 1:**
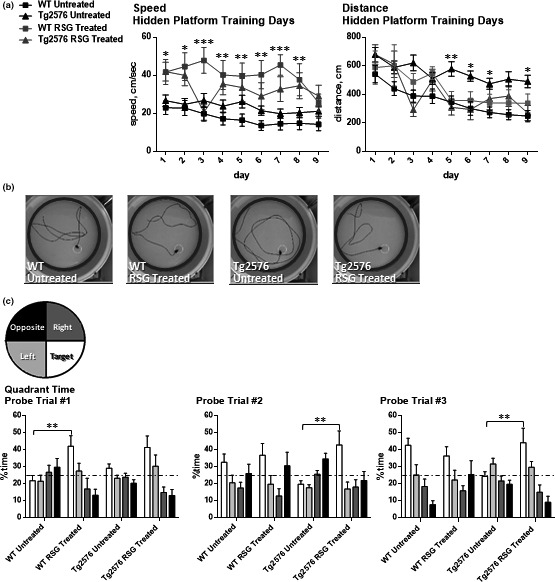
PPARγ agonism restores navigational learning and memory in 9MO Tg2576 mice. Spatial navigation memory was assessed using Morris water maze task. (a) Distance traveled to the hidden platform over nine training days was analyzed because repeated‐measures two‐way ANOVA determined that there were significant differences in swim speed between groups [Day *F*(8, 512) = 4.13; *p* < .0001] [Group *F*(3,64) = 7.74; *p* = .0002] [Subjects *F*(64, 512) = 10.69; *p* < .0001] with no significant interaction detected [*F*(24,512) = 1.515; *p* = .056]. Distance traveled to the hidden platform was significantly longer over several days in untreated Tg276 mice, whereas RSG‐treated AD and WT mice performed comparably during hidden platform trial training [Day *F*(8,512) = 11.4; *p* < .0001] [Group *F*(3, 64) = 10.82; *p* value < .0001] [Subjects *F*(64,512) = 3.45; *p* < .0001] with no significant interaction detected [*F*(24,512) = 1.22; *p* = .2189]. (b) Representative images tracing the path taken for each group during probe 2 trial. After 6 days of hidden platform training, untreated Tg2576 mice displayed aimless swimming patterns. (c) Two‐way ANOVA followed by Tukey's multiple comparisons test of probe trials determined that during probe trial 1, RSG‐treated WT performed better than untreated WT; during probe trials 2 and 3, in which the platform was removed, RSG‐treated Tg2576 mice spent more time exploring the target quadrant than untreated Tg2576. *p ≤ .05, **p ≤ .01, ***p ≤ .001, ****p ≤ .00001

We previously showed that one‐month PPARγ agonism with RSG improves hippocampus‐dependent associative learning and memory in 9MO Tg2576 as measured with the contextual (background) fear conditioning paradigm (Denner et al., 2012; Jahrling et al., 2014; Rodriguez‐Rivera et al., 2011). We therefore tested two cohorts of Tg2576 and WT littermates in the Morris water maze (Morris, 1984; Morris et al., 1982). After visible platform training, mice were subject to four daily trials to reach the hidden platform over nine consecutive days. During this training, the mice exhibited variable swim speeds; therefore, distance to the platform was used to test for spatial learning (Figure 1a). Two‐way repeated‐measures ANOVA indicated that select groups improved during the training in that there was a group and day effect but no interaction (Figure 1a). Untreated Tg2576 had impaired learning to spatially navigate the location of the hidden escape platform as indicated by the significantly longer distance to reach the escape platform on days 5–9. Tg2576 treated with RSG performed statistically equivalent to WT groups on days 1–9, indicating that Tg2576 undergoing PPARγ agonism treatment were now able to learn to spatially navigate to the escape platform. This is further supported by visual inspection of escape paths representative of each group (Figure 1b). Whereas untreated Tg2576 display what can be interpreted as a random circular search strategy; all other groups, including RSG‐treated Tg2576, appear to triangulate their position relative to the spatial cues placed external to the pool in order to find the hidden platform. Intermingled within the 9 days of hidden platform training were three probe trials (days 4, 7, and 10) in which the mice were placed in the pool and allowed to swim for 60 s; %time in each of four virtual quadrants was quantified. Two‐way ANOVA detected a significant interaction and treatment effect (Figure 1c). One‐sample *t* test against a theoretical mean of 25% did not detect that Tg2576 swam in the target quadrant preferentially during any of the probe trials. However, RSG‐treated Tg2576 swam in the target quadrant significantly more than chance level for all probe trials. By probe trial 2, Tukey's post hoc analysis detected that RSG‐treated Tg2576 performed significantly better than untreated Tg2576, indicating enhanced spatial memory. WT groups, untreated and treated, swam preferentially in the target quadrant as well and performed significantly better than untreated Tg2576. Quite interestingly, RSG treatment of WT mice revealed an enhancement of probe trial 1 performance compared with the untreated WT group (Figure 1c). By probe trials 2 and 3, this difference was not statistically significant. Thus, PPARγ agonism not only enhanced hippocampus‐dependent associative learning and memory in Tg2576 (Denner et al., 2012; Jahrling et al., 2014; Rodriguez‐Rivera et al., 2011) but also improved spatial learning and memory in 9MO Tg2576 and WT mice.

### 3.2 Novel object recognition

**FIGURE 2:**
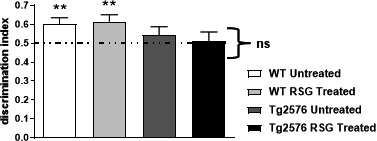
Novel object recognition was not rescued with RSG treatment utilizing a four‐hour retention interval between object familiarization and novel object introduction. WT groups (untreated, *n* = 17; RSG‐treated, *n* = 16) displayed significant preference for the novel object introduced for testing and were not enhanced with RSG treatment. Tg2576 groups (untreated, *n* = 10; RSG‐treated, *n* = 11) exhibited novel object recognition deficits that remained unchanged with RSG treatment. One‐sample *t* test against a theoretical mean of 0.5 determined that WT discrimination index was significantly different and that of Tg2576 was not. **p ≤ .01. Discrimination indices were calculated by dividing novel object exploration time by the sum of exploration time of the novel and familiar object

We previously demonstrated that 5MO Tg2576 exhibit object recognition deficits when we tested intermediate‐ and long‐term memory by using a 4‐ and 24‐hr retention interval: the interval between exploring two identical objects and when one of them was replaced with a novel object (Taglialatela et al., 2009). At 5MO, these deficits were ameliorated when the animals were acutely treated with the calcineurin (CaN) inhibitor, FK506. FK506 also reversed 5MO Tg2576 hippocampus‐dependent associative learning deficits as measured in the contextual fear conditioning task (Dineley et al., 2007). Since CaN inhibition did not improve contextual fear conditioning at 9MO (data not shown) but PPARγ agonism did (Denner et al., 2012; Jahrling et al., 2014; Rodriguez‐Rivera et al., 2011), we tested the hypothesis that RSG would ameliorate object recognition deficits in 9MO Tg2576. Object discrimination ratios revealed WT subjects explored the novel object more than the familiar object 4 hr after training, regardless of treatment. However, both untreated and RSG‐treated Tg2576 mice did not exhibit a preference for the novel object and explored the familiar and novel objects equally during testing. Therefore, PPARγ agonism did not rescue Tg2576 deficits in object recognition at 9MO (Figure 2).

### 3.3 Foreground fear conditioning

Previous work in our laboratory utilized background fear conditioning to reveal hippocampus‐dependent memory deficits in the Tg2576 model of AD that are reversed with RSG treatment. In this study, we asked whether 9MO Tg2576 mice exhibit a deficit in foreground fear conditioning in addition to the well‐established impairment in background fear conditioning. Foreground fear conditioning is very similar to background fear conditioning except there is no delivery of a discrete auditory stimulus with each 2‐s footshock. Contextual memory was tested 24 hr later by placing subjects back in the training environment without footshock exposure and freezing behavior quantified.

**FIGURE 3:**
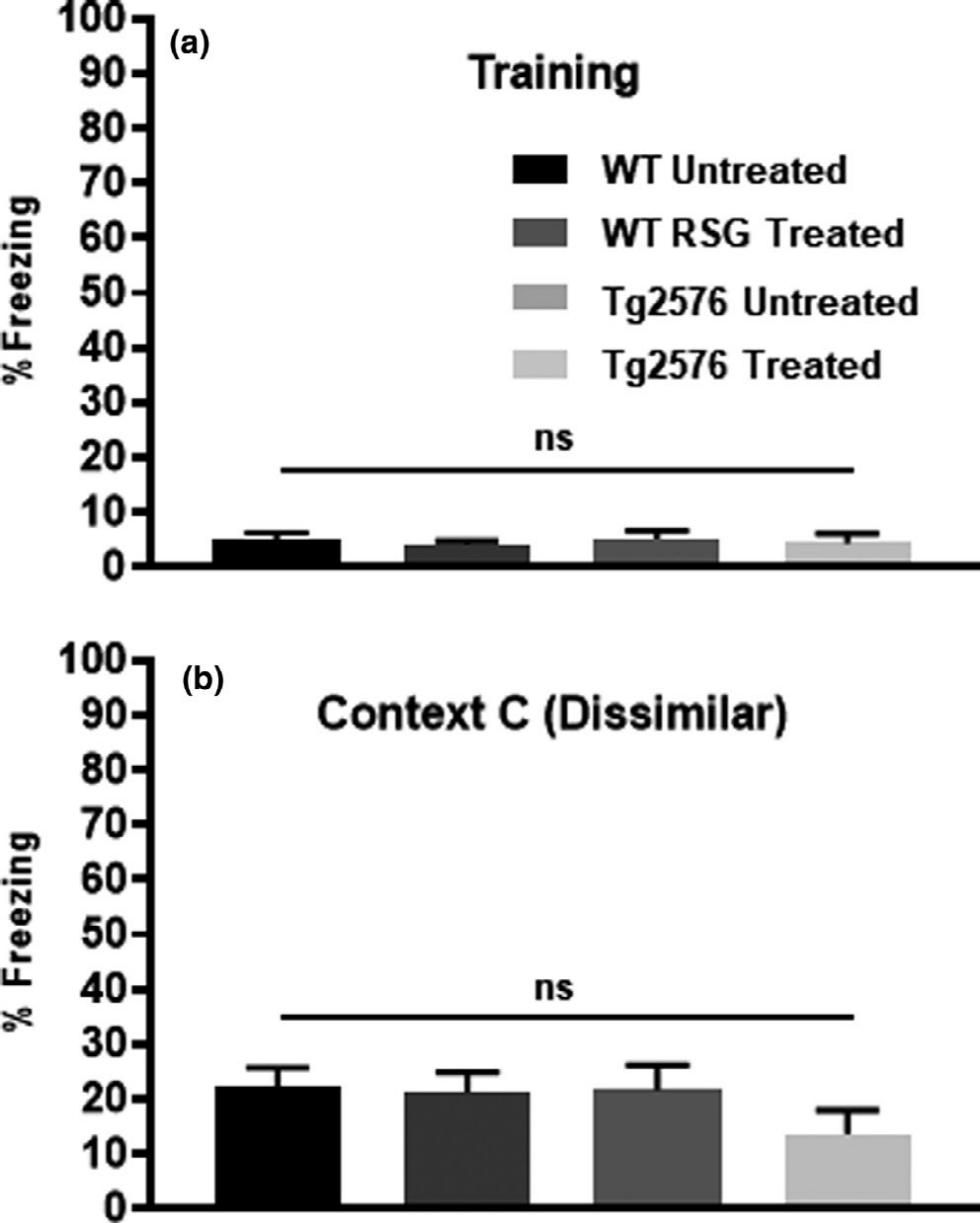
Freezing during training in Context A (a) and in the dissimilar Context C (b) was not altered between untreated and RSG‐treated groups. Training: *F*(3,63) = 0.144, *p* = .94. Context C: *F*(3,63) = 0.143, *p* = .24

During training, Tg2576 and age‐matched controls explored the context equally prior to the first footshock and freezing behavior ensued with delivery of each footshock (Figure 3a), as previously reported (Dineley et al., 2002, 2007; Rodriguez‐Rivera et al., 2011). During testing, Tg2576 mice froze significantly less than WT littermates. With freezing as a metric of learned behavior, 9MO Tg2576 mice are impaired in foreground fear conditioning learning and memory (Figure 3b).

### 3.4 Contextual fear discrimination

Similarities have been identified between foreground fear conditioning contextual memory and fear conditioning context discrimination circuitry (Huckleberry et al., 2016). Since 9MO Tg2576 are less able to associate footshocks with context in the foreground fear conditioning paradigm compared with WT littermates, we next asked whether Tg2576 mice are also able to discriminate between highly similar shock and safe contexts in the contextual fear discrimination paradigm, and if not, does RSG PPARγ agonism improve performance?

To validate that baseline exploratory behavior was unaffected by genotype or treatment, we compared total freezing in the training Context A prior to the first footshock on the training day 0 (Figure 3a). We also assessed freezing behavior in the completely dissimilar Context C on day 1 after testing in Context A and Context B (Figure 3b). There were no differences in total freezing among groups during seconds 1–178 of training in Context A, as well as during exposure to the dissimilar Context C on test day 1.

**FIGURE 4:**
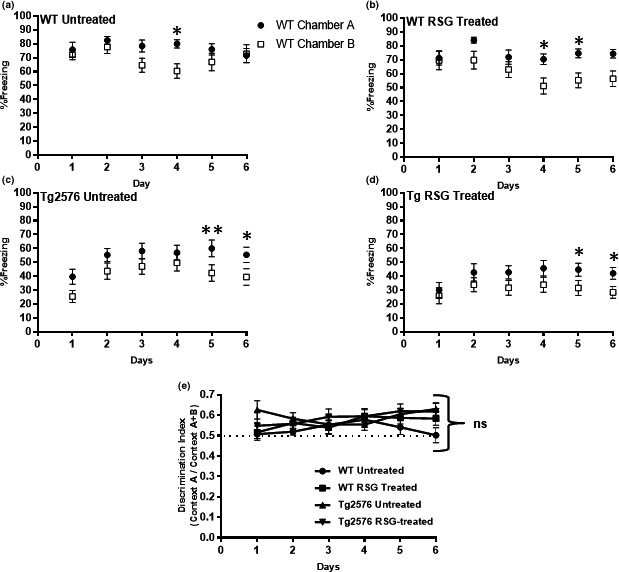
RSG improved context discrimination in 9MO WT. Figures represent total freezing across days in Context A and Context B for WT‐untreated (a), WT RSG‐treated (b), Tg2576‐untreated (c), and Tg2576 RSG‐treated (d) mice. Two‐way repeated‐measures ANOVA and Tukey's post hoc multiple comparisons of freezing data revealed that untreated and RSG‐treated WT first discriminate between aversive and highly similar contexts on day 4; RSG‐treated WT continued to discriminate, indicating PPARγ agonism enhanced context discrimination. Tg2576, either untreated or RSG‐treated, did not discriminate between Context A and Context B until day 5. Two‐way repeated‐measures ANOVA grouped analysis found a significant effect of testing day and genotype/treatment. Testing days: *F*(3.0,386) = 3.7, *p* = .01; genotype/treatment: *F*(7,130) = 33.4, *p* < .0001. Subject factor indicated that two‐way repeated‐measures ANOVA was an appropriate statistical test: *F*(130,650) = 2.3, *p* < .0001. No interaction found: *F*(35,650) = 1.4, *p* = .06. *p ≤ .05, **p ≤ .01. Grouped analysis of discrimination ratios (E) using two‐way repeated‐measures ANOVA revealed no difference between untreated and RSG‐treated WT or Tg2576 groups. Testing days: *F*(3.5,227.5) = 0.89, *p* = .46; genotype/treatment: *F*(3,65) = 2.45, *p* = .07; interaction: *F*(15,325) = 0.78, *p* = .70. Subject factor indicated that two‐way repeated‐measures ANOVA was an appropriate statistical test: *F*(65,325) = 1.4, *p* = .03. Discrimination ratios were calculated by dividing total freezing in Context A by the sum of freezing in Contexts A and B

**FIGURE 5:**
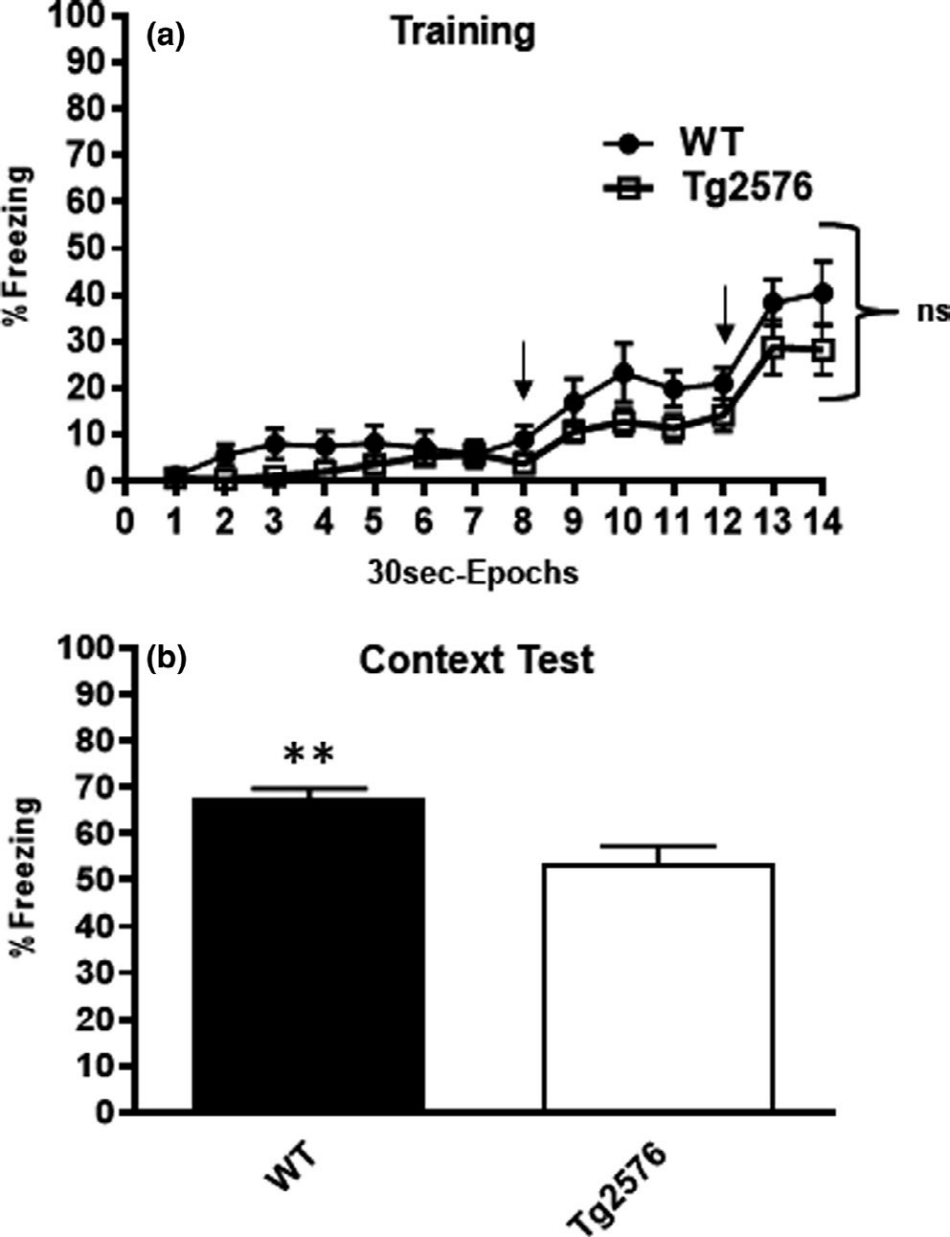
Tg2576 AD mice exhibit deficits in foreground contextual fear conditioning compared with age‐matched controls. Performance in foreground training (a) and contextual test 24 hr later (b). During training, both groups explored the novel context equally, while freezing begins to increase after each aversive stimulus at 240 s and 360 s, as indicated by arrows. Repeated‐measures two‐way (30 s epochs × genotype) ANOVA of freezing behavior during the 7‐min training session detected no difference in freezing behavior between the two genotypes [*F*(1,31) = 3.4; *p* = .075]. However, there was a significant epoch effect in that freezing increased following the first and second footshock [*F*(3.316,102.8) = 29.8; *p* < .0001]. 24 hr later, both groups were placed back into the training chamber to measure freezing to the shock context as a proxy for contextual fear memory. 9MO Tg2576 mice froze significantly less than WT littermates (*p* = .307; unpaired two‐tailed *t* test)

Sidak's two‐way ANOVA of %freezing behavior (Figure 4) found that WT groups (RSG‐treated and untreated) exhibited generalized fear as measured by freezing in both Context A and B during the first 3 days of testing (Figure 5a,b). By day 4, WT mice froze significantly less in the safe Context B compared with the shock Context A. Of note is that the WT RSG group continued to discriminate on day 5, while untreated WT mice were unable to discriminate on the final 2 days of testing, suggesting that RSG improved aged WT context discrimination. In contrast, Tg2576 groups (RSG‐treated and untreated) significantly discriminated on days 5 and 6 (Figure 5c,d), indicating that RSG had no effect on Tg2576 context discrimination. Grouped analysis using two‐way repeated‐measures ANOVA of freezing behavior found a significant effect of testing day and genotype/treatment.

Group analysis of daily discrimination ratios did not find any significant difference between groups throughout testing (Figure 5e). In summary, 9MO Tg2576 mice are impaired in both background and foreground contextual fear conditioning and the deficit in foreground fear conditioning is consistent with observed deficits in contextual discrimination fear conditioning. Whereas RSG improved WT context discrimination performance, PPARγ agonism had no effect on Tg2576 performance. RSG effect on 9MO WT context discrimination was reported previously (Cortez et al., 2019).

## 4 DISCUSSION

The current study extends previous work that established PPARγ agonism with the T2D drug RSG recues 9MO Tg2576 hippocampus‐dependent associative learning and memory using the background fear conditioning paradigm (Denner et al., 2012; Jahrling et al., 2014; Rodriguez‐Rivera et al., 2011). To support the involvement of dorsal hippocampus neurocircuitry, we previously determined that RSG normalized dentate gyrus intrinsic firing properties and presynaptic entorhinal cortex perforant path input to the dentate gyrus (Nenov et al., 2014, 2015). In this study, we attempted to expand our studies to test RSG effect on cognitive tasks that are heavily dependent on hippocampal function (spatial navigation in the MWM) and those that rely less on hippocampus (NOR) or test more subtle functions of the hippocampus (context discrimination). The evidence suggests that in the Tg2576 model, RSG treatment improves hippocampus‐dependent spatial memory in addition to hippocampus‐dependent associative fear memory. RSG did not improve object recognition that is less dependent upon hippocampus function. We will also argue that, based on hippocampus neurocircuitry underlying these neurobehaviors, dorsal hippocampus neurocircuitry is strongly implicated in being most sensitive to PPARγ agonism in the Tg2576 mouse model for AD.

Object recognition tests an animal's ability to recognize novelty after familiarization to two identical objects in a fixed position using designated retention intervals (Antunes & Biala, 2012; Taglialatela et al., 2009). Lesions that encompass the ventral hippocampus interfere with recognition memory, whereas those that are limited to the dorsal hippocampus have no effect (Broadbent et al., 2004). We previously published that 5MO Tg2576 mice exhibit NOR deficits using 4‐ and 24‐hr retention intervals that were ameliorated with CaN inhibition (Taglialatela et al., 2009). Using similar methods, we asked whether PPARγ agonism with RSG would restore object recognition after an intermediate‐term memory retention interval (4 hr). One‐month treatment with RSG from 8 to 9MO did not improve NOR performance in Tg2576 mice using a 4‐hr retention interval. In contrast, using the J20 AD mouse model, one‐month RSG treatment from 9 to 10MO restored 24‐hr NOR memory, suggesting that RSG intervention may be specific to long‐term NOR memory (Escribano et al., 2010). Interestingly, NOR deficits from hippocampal lesions that encompass dorsal *and* ventral hippocampus are associated with longer retention intervals (Broadbent et al., 2004; Hammond et al., 2004), indicating that the 4‐hr NOR retention interval used here did not fully engage the hippocampus and implicates parahippocampal involvement (Aggleton et al., 2010; Hammond et al., 2004).

Historically, the dorsal hippocampus and ventral hippocampus have been distinguished through differences in anatomical connectivity and electrophysiological–behavioral correlates (Moser & Moser, 1998a, 1998b; Moser et al., 1995; Richmond et al., 1999). Early work showed that the dorsal hippocampus, which corresponds to the posterior hippocampus in primates, was crucial for spatial navigation within a specific context, whereas the ventral (anterior in primates) hippocampus processes contextual information related to stress, emotion, and affect (Eichenbaum, 2017; Giustino & Maren, 2015; Jin & Maren, 2015). These functions are supported by circuit connectivity in that the dorsal hippocampus communicates with cortical regions (e.g., prefrontal cortex, sensory association areas) involved in decision‐making and information processing, while the ventral hippocampus communicates with regions involved in emotion and stress (e.g., amygdala and hypothalamus). Behavioral data have accumulated that is generally consistent with this segregation, although several exceptions have prompted neuroscientists to posit an intermediate zone of the hippocampus that serves as a convergence node between the dorsal hippocampus spatial/contextual and ventral hippocampus emotional/reward information that translates cognitive information into motivation and action critical for survival (Fanselow & Dong, 2010). As will be elaborated upon, the learning and memory tasks probed in this study indicate that Tg2576 exhibit both dorsal and ventral hippocampal circuit deficits; however, the efficacy of RSG PPARγ agonism is restricted to cognitive domains that rely more heavily on dorsal hippocampus.

Using the protocol developed for detecting age‐dependent spatial learning and memory decline in Tg2576 mice (Westerman et al., 2002), we found that RSG‐treated Tg2576 mice performed well, as well as WT littermates, in learning the location of the hidden platform and executing similar navigation strategies, suggesting RSG treatment rescues dorsal hippocampus‐dependent spatial learning and memory in 9MO Tg2576 mice. In a similar study, Escribano et al. (2010) demonstrated that prolonged RSG treatment (9–13MO) also improved spatial navigation, learning, and memory in the more aggressive double APP mutant mouse (J20), yet one‐month treatment from 9 to 10MO was ineffective. Though it is difficult to generalize between AD mouse models, amelioration of spatial navigation deficits in the J20 mouse model with extended treatment beginning at 9MO and our finding that one‐month treatment between 8 and 9MO is effective for Tg2576 suggest that therapeutic windows exist that correlate with the extent of amyloid pathology. Possibly, similar treatment windows exist in human LOAD. Likewise, disease stage‐specific therapeutic windows may prevail for cognitive domains and mechanistic interventions.

During probe trial 1 in the MWM, WT mice treated with RSG performed better than untreated WT mice, suggesting that PPARγ agonism improved 9MO WT mice performance. The observation that untreated WT mice performed equivalently to treated WT mice in probe trials 2 and 3 indicates this was a learning effect. Indeed, we have found that 9MO WT mice treated with RSG improve in measurable learning parameters during acquisition of the context discrimination paradigm (Cortez et al., 2019). Since our previous publications demonstrated that WT mice do not exhibit peripheral or central insulin resistance at 9MO, it suggests that cognitive enhancement with PPARγ agonism in 9MO WT mice is mediated by pathways distinct from those that improve insulin sensitivity (Rodriguez‐Rivera et al., 2011; Velazquez et al., 2017).

To address how PPARγ agonism can improve cognition in the absence of insulin resistance, we and others have described neuronal signaling pathways modulated by PPARγ agonism that are not directly associated with glucose uptake and insulin sensitivity (Denner et al., 2012; Perez & Quintanilla, 2015). In particular, we found that PPARγ agonism induced nuclear PPARγ binding to phosphorylated and kinase‐active ERK, an upstream MAPK that is associated with hippocampal synaptic plasticity‐dependent cAMP response element binding protein/CREB binding protein (CREB/CBP) gene expression (Jahrling et al., 2014). Furthermore, increased cAMP response element gene expression by PPARγ agonism improved CREB/CBP‐dependent expression of genes related to neurotransmission, synaptic plasticity, learning, and memory (Denner et al., 2012; Nenov et al., 2014). PPARγ agonism may also improve energy metabolism by stimulating mitochondrial biogenesis (Colca et al., 2014). Therefore, it is possible that the trend for improvement in MWM probe trial 1 in this group is from a gene expression repertoire beyond insulin sensitivity, possibly pathways associated with synaptic plasticity, learning, and memory.

We previously showed that RSG reversed Tg2576 deficits in the hippocampus‐dependent background fear conditioning task. Background fear conditioning consists of discretely pairing an auditory‐conditioned stimulus (CS) with an aversive‐unconditioned stimulus (US) (Denner et al., 2012; Jahrling et al., 2014; Rodriguez‐Rivera et al., 2011). As such, subjects learn to associate the context with this CS‐US pairing. It is widely accepted that the dorsal hippocampus is responsible for this memory in that pretraining lesions produce deficits in background fear conditioning expression (Anagnostaras et al., 2001; Bast et al., 2003) (Chang et al., 2008; Maren et al., 1997; Phillips & LeDoux, 1994; Stiedl et al., 2000). In contrast, perturbing *either* the dorsal or ventral hippocampus prior to *foreground* fear conditioning training *or* retrieval impairs foreground fear conditioning expression (Bast et al., 2001, 2003; Rudy & Matus‐Amat, 2005; Schenberg & Oliveira, 2008). Interestingly, this effect extends to silencing adult‐born neurons in either the ventral or dorsal subgranular zone of the dentate gyrus (Huckleberry et al., 2018). Together, this suggests that both dorsal and ventral hippocampi are important for pure contextual learning and memory, whereas background fear conditioning depends more heavily on dorsal hippocampus.

In background fear conditioning, it is postulated that the discrete paired auditory CS masks context features associated with the US and forces the subject to rely on multimodal cues for contextual fear memory recall. In foreground fear conditioning, the animals are not distracted by the auditory CS, allowing association of multiple contextual cues with the US. Since dorsal hippocampus drives background fear conditioning, possibly spatial aspects of the fear conditioning context are more heavily relied upon than the ventral hippocampus‐processed emotional associations of the US when recalling the context in which training occurred, whereas in foreground fear conditioning, all hippocampal information processing is associated with the US. Taken together, 9MO Tg2576 mice exhibit deficits in background and foreground fear conditioning, suggesting that Tg2576 mice possess both dorsal and ventral hippocampus circuitry lesions. Dorsal hippocampus circuit impairment was additionally supported by the observed deficits in MWM spatial learning and memory. While we did not test whether RSG reversed foreground fear conditioning deficits, our results from context discrimination fear conditioning provide insight.

While lesions of either the dorsal or ventral hippocampus interfere with context discrimination fear conditioning (Frankland et al., 1998; McDonald et al., 2018), recent work has focused on the role of adult neurogenesis within the subgranular zone of the dentate gyrus. Multiple studies have established that adult hippocampal neurogenesis, in particular within the dorsal hippocampus, correlates with performance in context discrimination learning and memory (Frankland, 1998; Sahay, Scobie, et al., 2011; Sahay et al., 2011). In addition, exercise and genetic manipulations that promote proliferation and maturation of adult‐born hippocampal neurons have positive effects on context discrimination learning and memory (Cortez et al., 2019; Creer et al., 2010; Sahay, Scobie, et al., 2011; Wu et al., 2015). Conversely, genetic ablation or cranial irradiation that depletes adult neurogenesis diminishes performance in contextual fear conditioning tasks (Cortez et al., 2019; Danielson et al., 2016; Saxe et al., 2006). We previously showed that RSG improved context discrimination in 9MO WT mice, in part through amelioration of neuroinflammation and resurrection of the neurogenic niche (Cortez et al., 2019). In this study, we asked whether RSG affected 9MO Tg2576 performance in the context discrimination fear conditioning task in which animals are required to differentiate between overlapping contextual cues, for example, grid floor (Cortez et al., 2019).

Context discrimination is similar to foreground fear conditioning but more challenging since animals are required to differentiate between overlapping contextual cues, for example, grid floor (Cortez et al., 2019). Overall, we found that WT performed better than Tg2576 mice and RSG treatment had no effect on either group. Though not conclusive, the fact that *either* ventral or dorsal hippocampus lesions interrupt context discrimination and foreground fear conditioning combined with our observation that RSG improved learning and memory in the MWM and background fear conditioning that rely upon dorsal hippocampus, but not context discrimination fear conditioning, suggests that RSG treatment is capable of resurrecting cognition that relies on dorsal hippocampus but not ventral hippocampus.

Several clinical studies have tested the efficacy of insulin‐sensitizing TZDs in AD patients, mostly reporting failure to prevent or improve cognitive and functional decline in those suffering moderate‐to‐advanced AD. In contrast, those AD pilot clinical trials assessing TZDs in subjects with early‐stage disease have found cognitive benefit in subjects comorbid for insulin resistance or those that are APOE4‐negative (Craft, 2012; Gold et al., 2010; Hanyu et al., 2009; Risner et al., 2006; Sato et al., 2011; Watson et al., 2005). Interestingly, these studies reported that cognitive improvement was detected only when select cognitive domains, predominantly episodic in nature, were analyzed. This supports the notion that TZD treatment failure in large double‐blind placebo‐controlled study designs may be attributed to a lack of refinement in neuropsychological testing analyses. Nonetheless, a recent large epidemiological study revealed a sizable protective effect of long‐term TZD treatment in T2D (Heneka et al., 2015), suggesting that exploratory studies of TZDs for AD cognitive enhancement remain warranted.

In conclusion, this study elaborated on the cognitive domain deficits that arise from over production of amyloid beta in 9MO Tg2576 model of AD with concomitant insulin resistance. We demonstrated that PPARγ agonism with RSG alleviated a somewhat narrow range of hippocampus‐dependent cognitive deficits that rely upon dorsal hippocampus. Further, we provide data that specific age‐related learning and memory can be enhanced with the diabetes pharmacotherapy, RSG PPARγ agonism even in WT mice. Thus, we propose that RSG PPARγ agonism may benefit those with early AD‐related memory decline with insulin resistance and possibly during aging in general.

## CONFLICT OF INTEREST

The authors report no conflict of interest.

## AUTHOR CONTRIBUTION

KTD, CMH, and IC designed the study. CMH and IC performed the experiments and processed the data. KTD, CMH, and IC analyzed the data and prepared the figures. KTD and IC wrote the manuscript.

### Peer Review

The peer review history for this article is available at https://publons.com/publon/10.1002/brb3.1973.

## Data Availability

The data that support the findings of this study are available from the corresponding author upon reasonable request.
